# Signal transduction pathway profiling of individual tumor samples

**DOI:** 10.1186/1471-2105-6-163

**Published:** 2005-06-29

**Authors:** Thomas Breslin, Morten Krogh, Carsten Peterson, Carl Troein

**Affiliations:** 1 Complex Systems Division, Department of Theoretical Physics, University of Lund, Sölvegatan 14A, SE-223 62 Lund, Sweden

## Abstract

**Background:**

Signal transduction pathways convey information from the outside of the cell to transcription factors, which in turn regulate gene expression. Our objective is to analyze tumor gene expression data from microarrays in the context of such pathways.

**Results:**

We use pathways compiled from the TRANSPATH/TRANSFAC databases and the literature, and three publicly available cancer microarray data sets. Variation in pathway activity, across the samples, is gauged by the degree of correlation between downstream targets of a pathway. Two correlation scores are applied; one considers all pairs of downstream targets, and the other considers only pairs without common transcription factors. Several pathways are found to be differentially active in the data sets using these scores. Moreover, we devise a score for pathway activity in individual samples, based on the average expression value of the downstream targets. Statistical significance is assigned to the scores using permutation of genes as null model. Hence, for individual samples, the status of a pathway is given as a sign, + or -, and a *p*-value. This approach defines a projection of high-dimensional gene expression data onto low-dimensional pathway activity scores. For each dataset and many pathways we find a much larger number of significant samples than expected by chance. Finally, we find that several sample-wise pathway activities are significantly associated with clinical classifications of the samples.

**Conclusion:**

This study shows that it is feasible to infer signal transduction pathway activity, in individual samples, from gene expression data. Furthermore, these pathway activities are biologically relevant in the three cancer data sets.

## Background

The interpretation of microarray data is facilitated by combining the data, or results of data analysis, with prior contextual knowledge, *e.g., *ontologies [1-4], pathways [5-7] and other annotation groups of interest [8]. By using prior knowledge about pathways, we aim at inferring cellular signaling pathway activity from tumor microarray data, on a sample-by-sample basis. Furthermore, we examine whether the pathway activity of individual samples is associated with clinical classifications of the samples. Our approach is in sharp contrast to establishing pathways from gene expression data (see *e.g. *[9]). What we do here is to project gene expresson data onto prior knowledge, in this case established pathway databases.

**Figure 1:**
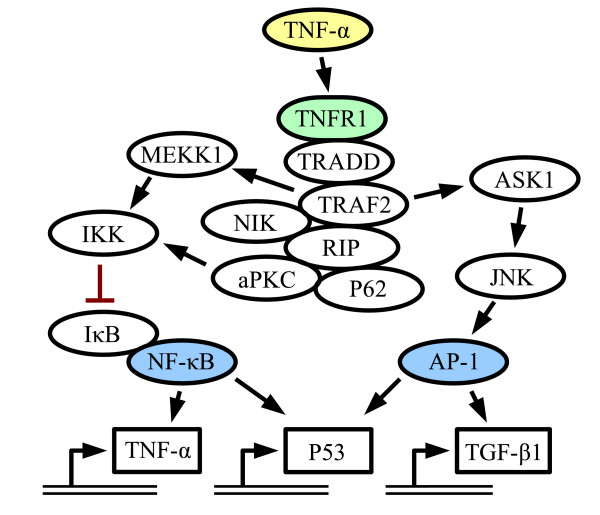
**Example pathway**. A simplified and partial view of the TNF-*α *pathway. A ligand (yellow) binds to a receptor (green) on the cell surface, triggering a cascade of events. Eventually, transcription factors (blue) activate or repress the expression of genes.

Signaling pathway activity scoring is a more direct measurement of biological processes than ontology mapping, which aims at finding over-representation of genes in various groups of contextual annotation. A cellular signaling pathway (see Fig. 1) is composed of a series of signaling molecules that convey information, typically from the outside of the cell to the nucleus. The initial step consists of extracellular signaling molecules, ligands, that activate receptors of the cell. These receptors then initiate intracellular signaling events, which eventually regulate the activity of various transcription factors. These transcription factors, in turn, regulate the expression levels of various genes, termed downstream targets of the pathway.

To characterize pathway activity, it would be desirable to have both proteomic and gene expression data. Gene expression data alone is not sufficient for assessing protein concentrations [10] and post-translational modifications of proteins. In the absence of proteomic data, one is thus forced to rely on aspects of the pathway that are detectable at the mRNA level. The foremost candidate for this is the downstream targets, which we will focus on here. It is of course also possible that the mRNA levels of effector proteins in a pathway change due to altered pathway activity. However, such effects are outside the scope of this paper. A further complication is that many pathways overlap, both in terms of having common transcription factors, and in terms of distinct transcription factors having common downstream targets. Our methods will not be able to distinguish very similar pathways, and in some sense this problem can be seen as a result of the ambiguities that follow when the full protein network is partitioned into separate pathways.

Cellular signaling pathways are subject to intense research, and current knowledge is compiled into databases such as STKE [11], TRANSPATH [12] and TRANSFAC [13]. These databases do not yet account for all pathways or transcription factors, but develop over time. Most pathway information utilized in this work is collected from TRANSPATH/TRANSFAC, where information about transcription factors and downstream targets is readily available. The only exception is the estrogen receptor pathway, which is taken from [14]. We analyze three microarray data sets in this study: Two breast cancer data sets [15,16], and one leukemia data set [17].

For the three data sets, we assess pathway activity from two related, but different, points of view. The first is to examine which pathways behave in a coherent way across the entire data set, *i.e., *which pathways have significantly co-expressed downstream targets. This is done both with and without accounting for the fact that downstream targets of a single transcription factor are correlated irrespective of pathway behavior. The second point of view is to assess pathway activity of individual samples, relative to the other samples in the same experiment, yielding an active or inactive status for each pathway in each sample. Finally, we relate the sample-wise pathway activity to clinical classifications of samples by way of contingency tables. Several pathways are found to be highly predictive of the clinical classifications.

## Results

In this section we assess variation in transcription factor and pathway activity across the samples. We then proceed to probe pathway activity in individual samples. Finally, we study the association between pathway activity and the clinical classifications of the samples.

### Co-expression of the downstream targets of a transcription factor

As a prelude to the study of pathways, we quantified the degree of correlation among downstream targets of single transcription factors. For this purpose we used the *Group Correlation Score *defined in Methods. The *p*-values were calculated using random reshuffling of the genes. Table 2 shows the most significant transcription factors in the van 't Veer data set. We see, as expected, that several transcription factors have significantly correlated downstream targets. For the data set of Sotiriou *et al., 8 *out of 42 transcription factors have a *p*-value below 0.1, and for the Golub *et al. *data set, the corresponding numbers are 6 out of 39. Among these three data sets, the *p*-values are noticeably better for data sets with more samples and genes. With this in mind we conclude that the downstream targets of transcription factors are co-expressed in the these data sets, albeit not to a high degree. The full lists for all 3 data sets can be found in Additional file 1.

**Table 1:** Transcription factor significance. The 15 most significant transcription factors (TF) in the van 't Veer *et al. *[15] data set, and their number of downstream targets (DT). *p*-values are based on the Group Correlation Score. In all 54 TFs were studied for this data set. ER pathway notation as in Table 1.

TF	# of DT	*p*-value
NF-*κ*B	19	4e-04
RelA	11	8e-04
ER Complex(i)	50	2e-03
NF-*κ*B1	10	2e-03
STAT1*α*	2	6e-03
C/EBP*α*	21	7e-03
ER-induced (v)	5	7e-03
ER	77	8e-03
STAT6	6	8e-03
ER(v)	7	2e-02
GATA-1	7	2e-02
Elk-1	4	6e-02
c-Rel	3	6e-02
STAT4	3	7e-02
SMAD-3	6	7e-02

### Co-expression of the downstream targets of a pathway

Here we use the same *Group Correlation Score *as above, applied to the downstream targets of entire pathways rather than those of individual transcription factors. Table 1 shows the results for the van 't Veer data set, where 21 out of 29 pathways have a *p*-value below 0.05, which is significantly more than expected by chance. However, many of the downstream targets have common transcription factors, which might be the major cause of the co-expression. To eliminate such a contribution we used the*Exclusive Group Correlation Score*, which considers only pairs of downstream targets lacking common transcription factors. The *p*-values for the *Exclusive Group Correlation Score *are also shown in Table 1. Although these *p*-values are larger, they are still significant; out of 20 pathways with more than one transcription factor, 12 have a *Exclusive Group Correlation Score p*-value below 0.05, which is still more than expected by chance. We conclude that the co-expression of downstream targets in a pathway can only in part be explained by the genes having common transcription factors. This co-expression at the pathway level justifies the view of pathways as functional units. Similar tables for the two other data sets are shown in Additional file 1. Both data sets have smaller *p*-values than expected by chance, albeit not as convincingly as the van 't Veer data set.

**Table 2:** Pathway significance. Pathways in the van 't Veer *et al. *[15] data set, ordered by significance and their number of transcription factors (TF) and downstream targets (DT). Also shown are Group Correlation Score (GCS) and Exclusive Group Correlation Score (EGCS) *p*-values. ER means both induced and repressed ER-pathway and (v) means that the pathway has been verified in a second experiment (see [14]).

Pathway	# of TF	# of DT	GCS *p*-value	EGCS *p*-value
IL-1	5	21	1e-04	4e-01
fMLP	9	27	4e-04	1e-01
TLR4	9	41	9e-04	1e-03
EDAR	6	41	3e-03	5e-02
ER-induced	1	50	3e-03	n/a
RANK	6	41	3e-03	5e-02
Oncostatin M	1	2	5e-03	n/a
PDGF	8	15	6e-03	2e-02
ER	1	77	7e-03	n/a
ER-induced(v)	1	5	7e-03	n/a
IL-4 – STAT6	1	6	8e-03	n/a
TGF-*β *network	7	23	1e-02	1e-02
EGF	12	53	1e-02	1e-02
ER (v)	1	7	2e-02	n/a
Insulin	7	45	2e-02	4e-02
VEGF	3	8	2e-02	2e-02
TNF-*α*	8	61	2e-02	5e-02
TPO	6	10	3e-02	2e-02
PRL	6	10	3e-02	2e-02
IFN	6	10	3e-02	2e-02
IL-10	2	7	5e-02	5e-02
IL-12 – STAT4	1	3	7e-02	n/a
c-Kit	4	87	8e-02	6e-02
ER-repressed	1	27	1e-01	n/a
B-cell antigen receptor	4	10	3e-01	3e-01
T-cell antigen receptor	4	10	3e-01	3e-01
Wnt pathway	2	8	4e-01	3e-01
ER-repressed (v)	1	2	6e-01	n/a
IL-2 – STAT5	2	4	8e-01	7e-01

### Pathway assignments for individual samples

After having established that downstream target genes are co-expressed in some pathways, we proceeded to study the status of pathway activity in individual samples. To this end we employed the *Group Sample Score*, which for each pathway designates every sample in a data set as either active or inactive, with an associated *p*-value.

Table 3 shows *p*-values and pathway activity status, for six samples in the van 't Veer data set. For example, in the first sample the RANK pathway is designated as inactive with a *p*-value of 0.004, whereas the inactiveness of the ER-induced pathway cannot be considered significant. The full tables for all samples and pathways in all 3 data sets are provided in Additional file 1.

**Table 3:** Sample pathway activity. The individual sample pathway activity *p*-values and sign for each pathway and six of the van 't Veer breast cancer samples. Bold face indicates significant *(i.e. p*-value ≤ 0.05) pathway activity in the sample. ER notation as in Table 1.

	1	2	3	4	5	6
ER-induced	0.7066(-)	**0.0000**(+)	0.4822(+)	0.1648(+)	0.4106(+)	**0.0072**(+)
EDAR	**0.0052**(-)	0.1514(-)	0.1294(-)	0.8566(+)	**0.0010**(-)	**0.0008**(-)
IL-1	**0.0074**(-)	**0.0416**(-)	**0.0158**(-)	0.3848(+)	**0.0088**(-)	**0.0002**(-)
RANK	**0.0058**(-)	0.1506(-)	0.1340(-)	0.8598(+)	**0.0022**(-)	**0.0008**(-)
TNF-*α*	**0.0014**(-)	0.1744(-)	0.2596(-)	0.4044(+)	**0.0030**(-)	**0.0006**(-)
EGF network	0.2868(-)	0.3030(-)	0.4522(-)	0.9360(-)	0.2138(-)	**0.0000**(-)
ER	0.9994(-)	**0.0002**(+)	0.3074(+)	0.7624(+)	0.7886(-)	**0.0002**(+)
ER-induced (v)	0.4940(-)	**0.0136**(+)	0.1670(+)	0.3060(+)	0.3310(+)	0.3750(+)
TLR4	0.1584(-)	0.2572(-)	0.2624(-)	0.7778(+)	0.1856(-)	**0.0000**(-)
fMLP	**0.0122**(-)	**0.0432**(-)	**0.0004**(-)	0.7188(+)	**0.0362**(-)	**0.0268**(-)
Insulin	0.9274(-)	**0.0182**(-)	0.3926(-)	0.6116(+)	0.7898(+)	**0.0172**(-)
ER (v)	0.9382(-)	**0.0084**(+)	0.0680(+)	0.0806(+)	0.3266(-)	0.0802(+)
TGFβ network	0.1102(+)	0.7054(+)	0.8274(-)	0.3528(-)	0.7902(-)	0.2550(-)
c-Kit	0.2892(-)	**0.0282**(-)	0.3000(-)	0.7884(-)	**0.0470**(-)	0.2398(-)
ER-repressed (v)	0.3518(+)	0.0566(+)	0.0588(+)	**0.0426**(+)	**0.0034**(-)	0.0536(+)
VEGF	0.6526(-)	0.3174(+)	0.4388(-)	0.9372(-)	0.8526(-)	0.8608(-)
IL-10	0.3984(-)	0.5984(+)	0.8164(-)	0.8188(+)	0.2442(-)	0.7698(+)
IFN	0.3934(-)	0.7678(+)	0.4792(-)	0.7824(+)	0.5702(-)	0.7430(-)
PRL	0.3984(-)	0.7726(+)	0.4794(-)	0.7840(+)	0.5782(-)	0.7344(-)
TPO	0.3852(-)	0.7500(+)	0.4736(-)	0.8056(+)	0.5588(-)	0.7388(-)
PDGF	0.9488(+)	0.4568(-)	0.6370(-)	0.5418(-)	0.6680(+)	0.4060(-)
Oncostatin M	0.2746(-)	0.2986(-)	0.1848(-)	0.1428(+)	0.7450(+)	**0.0046**(-)
T-cell antigen receptor	0.2092(-)	0.1006(-)	**0.0094**(-)	0.9426(+)	0.6404(-)	0.3304(+)
B-cell antigen receptor	0.1978(-)	0.1040(-)	**0.0142**(-)	0.9484(+)	0.6508(-)	0.3222(+)
IL-12 – STAT4	0.3458(+)	0.4402(+)	0.8902(+)	0.6424(+)	0.1982(-)	0.9878(-)
ER-repressed	0.5724(+)	0.4596(+)	0.3936(+)	0.1784(-)	0.0830(-)	**0.0158**(+)
IL-2 – STAT5	0.1816(-)	0.8828(-)	0.4774(-)	0.7768(-)	0.3486(+)	**0.0376**(-)
IL-4 – STAT6	0.8268(-)	0.1670(-)	0.9212(-)	0.3412(-)	0.1430(+)	0.1510(-)
Wnt	0.8484(-)	0.1054(-)	0.0878(-)	0.5868(-)	0.3756(-)	0.5084(-)

Table 4 shows the number of samples that are active and inactive at the 5% level, for every pathway in the van 't Veer data set. The table also contains the family-wise *p*-value, defined in Methods, which gives the probability of observing at least this total number of significant samples for a pathway. The family-wise *p*-value assumes that the samples are independent, which is only approximately true since the mean expression value of a gene across all samples is zero. Corresponding tables for the two other data sets can be found in Additional file 1. We note that the most significant pathways according to this measure are mostly the same as with the correlation based scores, although the *p*-values are numerically different.

**Table 4:** Pathway significance. This table shows the number of samples (out of 117) in the van 't Veer data set with pathway status active (+) or inactive (-) and with *Group Sample Score p*-value ≤ 0.05. Also shown are the corresponding family-wise *p*-values. ER notation as in Table 1.

Pathway	+	-	family-wise *p*-value
ER-induced	30	37	2e-55
EDAR	30	30	6e-46
IL-1	25	34	1e-44
RANK	29	30	1e-44
TNF-*α*	29	29	2e-43
EGF network	29	29	2e-43
ER	28	29	4e-42
ER-induced(v)	21	34	1e-39
TLR4	28	27	1e-39
fMLP	24	31	1e-39
Insulin	22	21	6e-26
ER (v)	17	21	6e-21
TGF*β *network	20	18	6e-21
c-Kit	20	14	3e-17
ER-repressed (v)	11	17	4e-12
VEGF	11	15	1e-10
IL-10	12	13	7e-10
IFN	11	14	7e-10
PRL	11	14	7e-10
TPO	10	14	3e-09
PDGF	9	12	4e-07
Oncostatin M	6	15	4e-07
T-cell antigen receptor	11	8	6e-06
B-cell antigen receptor	11	7	2e-05
IL-12 – STAT4	7	9	2e-04
ER-repressed	7	6	6e-03
IL-2 – STAT5	5	7	1e-02
IL-4 – STAT6	5	3	2e-01
Wnt	4	4	2e-01

**Figure 2:**
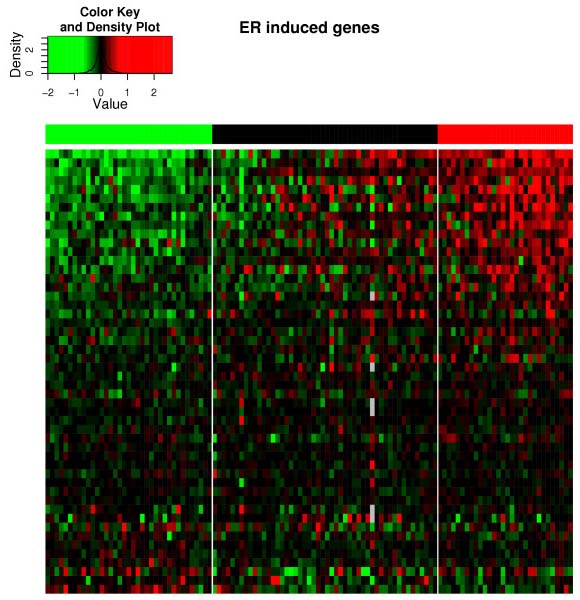
**ER-induced pathway heatmap**. Heatmap of the ER-induced pathway activity corresponding to Table 4. The vertical divisions correspond to samples where the ER-induced pathway is significantly upregulated (30 samples, red bar), its status undetermined (50 samples, black bar) or significantly downregulated (37 samples, green bar) respectively. The rows represent genes in the ER-induced pathway. The genes are shown in descending order according to scalar product with the vector ± (1 - *p*-value) for each sample, where the sign is the sign of the pathway activity of that sample. The expression values are log ratios normalized as described in Methods. Red and green represent up- and down-regulation respectively, and the precise color scheme is illustrated in the color key.

**Figure 3:**
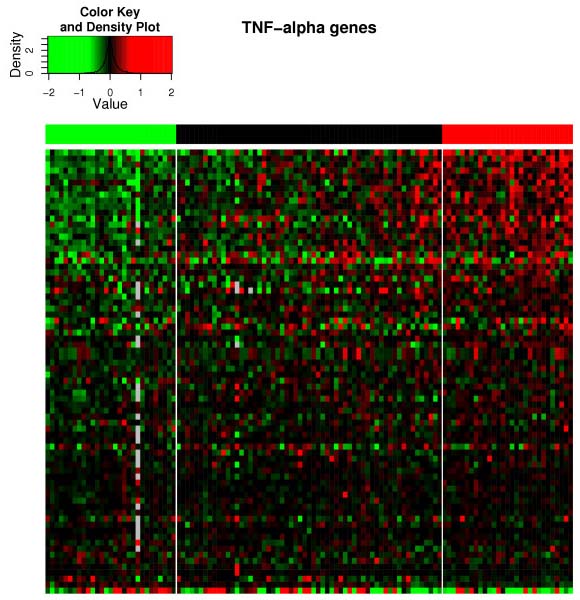
**TNF-*α *heatmap**. Heatmap of the TNF-*α *pathway activity corresponding to Table 4. Notations as in Figure 2.

In Figures 2 and 3, we show heatmaps for two of the pathways, ER-induced and TNF-*α *respectively. These two examples are illustrative; there is a substantial fraction of genes that are clearly upregulated in the active samples and downregulated in the inactive samples, as seen by the red upper right corner and green upper left corner. There are also many genes in the pathway that do not seem to be regulated, and even a few which act oppositely. The latter ones are possibly genes, that are repressed by the pathway. Similar figures for the entire genome (not shown) do not show large red or green upper corners, confirming the statistical analysis.

### Association between sample-wise pathway activity and clinical classifications

We analyzed the association between sample-wise pathway activity and clinical classifications using contingency tables. For every pathway and data set, we divided the samples into three groups: Samples where the pathway was active at a 5% significance level, samples where it was inactive at a 5% significance level, and insignificant samples referred to as undecided. For each data set, contingency tables of pathway activity versus clinical classifications were created, and *χ*^2 ^*p*-values were calculated.

In the data set of Golub *et al., *the only available clinical classification is tumor type, *i.e., *ALL or AML. Table 5 shows the contingencies for the Insulin pathway and the I1-1 pathway. Seven out of 29 pathways have contingency tables with a *χ*^2 ^*p*-value below 0.01.

**Table 5:** Contingency tables for the ALL/AML status versus the Insulin and IL-1 pathways in the leukemia data set of Golub *et al. *[17]. Active, non-active and undecided pathways are denoted +, - and U respectively.

	ALL	AML
Insulin pw(+)	1	5
Insulin pw(-)	15	0
Insulin pw(U)	11	6

*p*-value: 1e-05		

	ALL	AML

IL-1 pw(+)	0	6
IL-1 pw(-)	19	0
IL-1 pw(U)	8	5

*p*-value: 5e-04		

For the breast cancer data set of van 't Veer *et al., *we investigated six clinical classifications: metastasis status (0), estrogen receptor status (20), progesterone receptor status (12), lymph node status (12), BRCA mutations (15) and histological grade (8). The numbers in parentheses refer to the number of significant contingency tables at the 0.01 level. The total number of pathways was again 29. For metastasis status, only 97 out of the 117 samples were labeled in the original data set, and this may contribute to the low degree of association between this clinical classification and pathway activity. However, similar results were obtained for the data set of Sotiriou *et al., *which indicates that it may be difficult to obtain any association between the pathways analyzed in this work and breast cancer metastasis status. Table 6 shows the contingency between estrogen receptor status and the ER-induced pathway. As expected, there is a strong association between presence of the estrogen receptor protein, and the activity status of the ER-induced pathway. Somewhat more surprisingly, there are also strong associations between ER status and many other pathways. Similar results are obtained for the data set of Sotiriou *et al., *but with fewer significant associations.

**Table 6:** Contingency tables of estrogen receptor protein (binned at three levels: 0, 5–50, 60–100) versus the ER-induced and RANK pathways in the breast cancer data set of van't Veer *et al. *[15]. Same notation as in Table 5.

	ERp low	ERp med.	ERp high
ER-ind pw(+)	0	5	25
ER-ind pw(-)	31	3	3
ER-ind pw(U)	8	16	26

*p*-value: 2e-14			

	ERp low	ERp med.	ERp high

RANK pw(+)	25	2	2
RANK pw(-)	1	5	24
RANK pw(U)	13	17	28

*p*-value: 1e-11			

A general tendency of the contingency table analysis is illustrated in Table 7. Lowering the pathway activity *p*-value cutoff makes the association to clinical classifications more specific but less sensitive. The complete set of contingency tables for all three data sets can be found in Additional file 1.

**Table 7:** Contingency table of lymphocytic infiltration status versus the IL-12/STAT4 pathway in the van't Veer data set. The upper and lower tables are obtained with a pathway activity cutoff at 0.05 and 0.1 respectively. Same notation as in Table 5.

*cutoff: 0.05*	L+	L-
IL-12/STAT4 pw(+)	0	7
IL-12/STAT4 pw(-)	9	0
IL-12/STAT4 pw(U)	80	21

*p*-value: 2e-05		

*cutoff: 0.1*	L+	L-

IL-12/STAT4 pw(+)	2	8
IL-12/STAT4 pw(-)	13	0
IL-12/STAT4 pw(U)	74	20

*p*-value: 2e-05		

## Conclusion

We have shown that downstream target genes of signal transduction pathways behave coherently in gene expression tumor data sets. First, we confirmed that downstream targets of transcription factors are correlated across samples. We then demonstrated that the same holds true for downstream targets of an entire pathway, even after discounting the correlations due to genes having a common transcription factor. The correlations for entire pathways were found to be more significant than those for individual transcription factors.

The presence of significant correlations confirms the expectation that gene expression is controlled by the activity of pathways. However, these correlations do not tell us in which samples a pathway is active or inactive. To reveal this, we devised the *Group Sample Score*. With this score we classified the samples into those where the pathway was significantly active, significantly inactive or undecided, respectively. As seen in Table 4, the number of significant samples is, for most pathways, much higher than the random expectation.

In many cases, the active/inactive pathway status was highly correlated with independent clinical classifications. This confirms the relevance of pathways for understanding of the underlying biology. Furthermore, the activity status of one or more pathways may be used to subdivide the samples into groups with distinct biological characteristics. Such a subdivision is feasible if, for instance, tumors of a certain clinical diagnosis are an agglomerate of several subtypes.

The *Group Sample Score *is natural if a pathway either induces all its downstream targets, or represses them. However, in most pathways some downstream genes are induced, while others are repressed. To account for a mixture of induction and repression, one should include a sign, or more generally a weight, to each term in the sum. Such a weight might even depend on the type of tissue and the environment. Since this information was not readily available for the studied pathways, all genes were weighted equally. Nevertheless, we obtained significant results, indicative of a dominant trend among the downstream genes. For the estrogen receptor (ER) pathway, we did have information about the sign, but instead of introducing a more general score for this pathway alone, we split the ER pathway into two parts, with induced and repressed genes, respectively. In the breast cancer data set of van 't Veer *et al. *[15], there were 50 genes in the induced part and 27 in the repressed. As seen in Tables 1 and 4, the ER-induced pathway was highly significant, whereas the repressed pathway was not. The full pathway was also highly significant, although to a lesser extent. The significance of the full pathway is thus due to the induced genes, which constitute a majority of the downstream targets. The situation is similar for other pathways and data sets.

It should be stressed that correlations, and the pathway activity status observed in a sample, are only defined relative to the other samples in the same data set. If a pathway were active in all samples, it would not show up in our significance test. The status of a pathway, as we define it, is given by the downstream genes, and the connection to ligands, receptors and other pathway components cannot be inferred from this analysis.

Table 2 shows that the most significant transcription factor in the breast cancer data set of van 't Veer *et al. *is NF-*κ*B. This transcription factor is also the most one in the leukemia data set of Golub *et al., *whereas NF-*κ*B1 is the most significant one in the data set of Sotirou *et al *Recently, NF-*κ*B has been shown to be involved in the transformation from benign to malignant cells in inflammation-associated cancers. Pikarsky *et al. *[18] demonstrate this in a mouse model of human hepatocellular carcinoma, where the inflammatory mediator tumor-necrosis factor-*α *(TNF-*α*) is shown to play an important role as an activator of NF-*κ*B. Greten *et al. *[19] find similar results in a mouse model of colitis-associated cancer.

Our current knowledge of pathways, and of downstream targets of transcription factors, is far from complete. However, we find that the results presented herein constitute a proof of concept for analyzing microarray gene expression in the context of signal transduction pathways.

## Methods

### Pathway information and UniGene clusters

Transcription factors for 23 pathways were extracted from TRANSPATH [12]. The downstream target genes of those transcription factors were obtained from TRANSFAC [13]. Since our study contains breast cancer data, we have augmented the pathway information with the Estrogen Receptor (ER) pathway compiled from [14], where 89 direct target genes were identified. 59 of them were induced by the ER complex and 30 were repressed. 8 out of the 89 genes were previously verified. We employ all six combinations of induced/repressed/all and verified/all, yielding 6 versions of the ER pathway. The 29 pathways employed are listed in Table 1. Downstream target genes were represented as UniGene IDs , using UniGene Hs build 171. For the analysis of the data sets, gene identifiers were converted into UniGene IDs and expression values of clones belonging to the same UniGene cluster were averaged.

### Data sets

The following three publicly available data sets were analyzed:

1. The breast cancer data set of van 't Veer *et al. *[15], consisting of samples from 117 patients, of which 46 developed metastases. After UniGene merging the data set contains 20663 genes.

2. The breast cancer data set of Sotiriou *et al. *[16], consisting of 99 samples of different clinical classifications, with 4878 genes after UniGene merging.

3. The leukemia data set of Golub *et al. *[17], derived from bone marrow samples from 38 patients, 27 of which were diagnosed with Acute Myeloid Leukemia (AML) and 11 with Acute Lymphoblastic Leukemia (ALL). After Uni Gene merging and removal of genes with no variance across the samples, 4701 genes remain.

### Normalization of microarray data

The data sets in [15,16] are given in the form of log ratios of expression values in the samples versus a reference. The data set in [17] is given in the form of Affymetrix average difference values. For the calculation of *Group Correlation Score *and *Exclusive Group Correlation Score *the affymetrix average difference values were logarithm transformed, since the Pearson correlation is very sensitive to single outlier samples. For the *Group Sample Score *the original differences were kept. We denote the expression value for gene *g *in sample *s *by *x*_*gs*_, with missing values allowed. We normalized the expression values in two steps. First, for each sample, the mean of all genes was subtracted, in order to ensure that no samples are up- or down-regulated on average. The transformed expression values satisfy:



Second, for each gene, the mean of all samples was subtracted from the expression values of that gene, yielding:



The second normalization implies that the expression value of a gene is measured relative to the same gene in other samples.

### Pearson correlation *p*-values

To determine if a group of downstream target genes is significantly co-expressed, a total score of the group is needed. Two scores were used here, both based on the Pearson correlation of a pair of genes *g *and *h*:



where the sums exclude missing values and  is the mean of expression values for gene *g*.

The *Group Correlation Score *is defined as the sum of squares of Pearson correlations among all pairs of genes in a group of genes:



where the sum runs over all genes in the group. The square ensures that both correlations and anti-correlations contribute to the score. We use the *Group Correlation Score *for the downstream target genes of a single transcription factor, as well as for those of an entire pathway.

The *Exclusive Group Correlation Score*, on the other hand, is only applicable for the downstream targets of a pathway. It is defined as



where the sum runs exclusively over pairs of genes *g *and *h *that do not share any transcription factor.

The *p*-value of a score is defined as the fraction of random cases, drawn under the null hypothesis, which achieve a higher score than the score tested. For both scores, *GCS *and *EGCS*, our null hypothesis is reshuffling of the genes on the microarray. This null hypothesis keeps the structure and overlap of all pathways fixed, but changes the identity of the genes.

### Pathway activity for individual samples

For each sample, *s*, and pathway, *PW*, the *Group Sample Score *is defined as follows:



where the sum runs over all downstream target genes of the pathway.

The null hypothesis is again reshuffling of the genes from the microarray. We are interested in pathways both with high and low scores. Hence, we consider the *p*-values for the score being higher (*p*_+_) and lower (*p*_-_) than random, respectively, and the final *p*-value is given by two times the smaller of these two *p*-values:

*p *= 2·min(*p*_+_, *p*_-_).

The pathway is said to be active (+) if *p*_+ _<*p*_-_, and inactive (-) otherwise.

### Family-wise *p*-value

If *N *independent hypotheses are tested simultaneously, the probability to obtain *K *or more *p*-values below *q *is given by a binomial distribution:



We refer to this probability as the *family-wise p-value*.

## Authors' contributions

TB, MK, and CT implemented the algorithms employed. All authors contributed conceptually to the methods presented herein, as well as to the preparation of the manuscript.

## Supplementary Material

Additional File 1 All additional files are in tab-delimited format. Tablel_Golub.csv Tablel_Sotirou.csv Table2_Golub.csv Table2_Sotirou.csv Table2_Veer.csv Table3_Golub.csv Table3_Sotirou.csv Table3_Veer.csv Table4_Golub.csv Table4_Sotirou.csv Contingency_Tables_Golub.csv Contingency_Tables_Sotirou.csv Contingency_Tables_Veer.csv Click here for file
